# Understanding the Relationships Between Fear of COVID‐19, Depression, Loneliness, and Life Satisfaction in Türkiye: Testing Mediation and Moderation Effects

**DOI:** 10.1002/nop2.70204

**Published:** 2025-03-26

**Authors:** Orhan Koçak, Murat Yıldırım, Orçun Muhammet Şimşek, Orhan Çevik

**Affiliations:** ^1^ Faculty of Health Sciences Istanbul University–Cerrahpaşa Istanbul Türkiye; ^2^ Department of Psychology Agri Ibrahim Cecen University Ağrı Türkiye; ^3^ Psychology Research Centre Khazar University Baku Azerbaijan; ^4^ Department of Social Work Istanbul Nisantasi University Istanbul Türkiye; ^5^ Institute of Graduate Studies Istanbul University–Cerrahpaşa Istanbul Türkiye

**Keywords:** depression, fear of COVID‐19, loneliness, satisfaction with life, Turkish youth

## Abstract

**Aim:**

This study examined the mediating roles of depression and various dimensions of loneliness (family, social, and romantic) in the associations between COVID‐19 fear and satisfaction with life. Additionally, the research investigated how the economic impact of COVID‐19, family income, gender, and marital status may moderate these associations.

**Design:**

A cross‐sectional descriptive design.

**Methods:**

An online survey was conducted in Türkiye. 1702 participants completed self‐report measures assessing COVID‐19 fear, depression, social, emotional, and romantic loneliness, and satisfaction with life.

**Results:**

The findings revealed positive associations between COVID‐19 fear and depression, along with negative relationships between COVID‐19 fear and family, social, and romantic loneliness. Depression was identified as a mediator in the relationship between COVID‐19 fear and the different components of loneliness. These loneliness components, in turn, influenced life satisfaction. Furthermore, the study found significant moderation effects related to the economic impact of COVID‐19 restrictions, family income, marital status, and gender.

**Conclusions:**

This study provides evidence about the psychological mechanisms linking COVID‐19 fear to life satisfaction. It highlights the mediating role of depression and the moderating effects of demographic and economic factors. The findings emphasise the need for targeted mental health interventions, particularly addressing the varied impact of COVID‐19 fear across different social and economic groups. This evidence can inform strategies aimed at improving life satisfaction during periods of social and economic disruption.

**Patient or Public Contribution:**

This study has no direct patient involvement in its design, conduct, or reporting. However, it contributes to public health by providing evidence about the psychological impacts of COVID‐19, which may inform future interventions and mental health support strategies.

## 1 Introduction

The COVID‐19 disease, initially identified in Wuhan, China, in December 2019, rapidly disseminated globally within a year. The World Health Organisation (WHO) declared the disease a pandemic in March 2020. According to the latest data from the World Health Organisation as of 24.12.2023, the cumulative worldwide COVID‐19 cases reported are 773,119,173 million, with 6,990,067 million recorded deaths. In Türkiye, there were 17,004,677 cases and 101,419 deaths from COVID‐19 by the same date (World Health Organization 2023). Pandemics have periodically emerged, claiming the lives of millions. The physical and psychological toll during the pandemic, as well as post‐traumatic stress disorders in its aftermath, have had enduring adverse effects on individuals (Armiya'u et al. 2022; Çağış and Yıldırım 2023; Rehman et al. 2023).

The quarantines, curfews, and closures of workplaces, schools, cinemas, theatres, and entertainment venues during the peak of the COVID‐19 pandemic have led to unprecedented situations and a decline in individuals' mental well‐being (Geçer and Yıldırım 2023). Beyond these challenges, the emergence of COVID‐19 fear and anxiety—fuelled by the absence of definitive treatment, vaccine efficacy speculation, and a rapid surge in unemployment rates—has heightened people's concerns about the future, significantly elevating levels of depression, anxiety, and stress (Akgül 2020; Clair et al. 2021; Green and Yıldırım 2022; Koçak et al. 2021). The psychological consequences caused by COVID‐19‐related issues have also negatively impacted the social, family, and romantic connections of individuals, leading to a decrease in life satisfaction. This study addresses how COVID‐19 fear affects life satisfaction, exploring its associations with depression, family emotional loneliness, social loneliness, romantic emotional loneliness, and overall life satisfaction in Türkiye.

### 1.1 COVID‐19 Fear, Depression and Loneliness

Increased mobility facilitated by advanced transportation has accelerated the rapid global spread of the COVID‐19 virus (Borkowski et al. 2021; Saha et al. 2020). The media and communication channels swiftly disseminated potential adverse effects and scenarios of COVID‐19, inducing widespread fear and concern among the population (Bendau et al. 2021; Choi et al. 2020; Liu and Liu 2020). Government‐imposed restrictions, economic challenges, the isolation of the elderly, the closure of schools hindering young people's socialisation, and uncertainties surrounding the pandemic have collectively heightened societal psychological distress, adversely impacting mental health (Abuselidze and Mamaladze 2020; Jain et al. 2020; Kumar et al. 2021; Lakhan et al. 2020). Studies have shown varying levels of COVID‐19 fear, with some indicating higher fear in the elderly and others in the young (Hyland et al. 2020; Koçak et al. 2021). Across numerous studies on the psychological impact of COVID‐19, it was found that the elderly, young people, workers, refugees, and individuals with chronic diseases are at higher risk of infection and experience greater psychological effects such as depression and stress (Koçak et al. 2021; Salari et al. 2020; Shevlin et al. 2020). This sets the stage for the following hypothesis.

Hypothesis 1 *There is a positive association between COVID‐19 fear and depression*.

Loneliness, a negative emotion characterised by a sense of social detachment and uncertainty, arises when there is a disparity between actual and expected social relationships (Goossens et al. 2009; Lim et al. 2020; Loades et al. 2020). It is a multifaceted phenomenon with different causes and consequences for individuals (Killgore et al. 2020a, 2020b; Lim et al. 2020). Loneliness is often categorised into social and emotional types, with the absence of a social network contributing to social loneliness and a lack of close interaction leading to emotional loneliness (Goossens et al. 2009). Loneliness can trigger additional problems (Clair et al. 2021; Donovan and Blazer 2020). Despite being perceived as temporary, loneliness is a distressing experience with potentially far‐reaching effects on life satisfaction (Bozorgpour and Salimi 2012; Esen and Siyez 2011).

Both social and emotional loneliness can adversely impact the psychological and physical health of individuals (Holt‐Lunstad et al. 2015; Strizhitskaya et al. 2021). The COVID‐19 pandemic‐related restrictions exacerbated physical loneliness due to increased physical and social isolation. Paradoxically, individuals' fear of COVID‐19 reduced emotional loneliness, strengthening their emotional ties to relatives, families, and romantic partners (Strizhitskaya et al. 2021). While loneliness is often associated with perceived solitude or a lack of social support (Hawkley and Cacioppo 2010), studies during the pandemic revealed an increase in perceived social support (Strizhitskaya et al. 2021; Luchetti et al. 2020).

Contrary to initial expectations, emotional loneliness during COVID‐19 was found to be lower than before the pandemic in some studies (Strizhitskaya et al. 2021). Additionally, other research suggested that the pandemic did not significantly affect overall loneliness and even increased perceived social support (Luchetti et al. 2020). Importantly, during active periods of the pandemic, individuals, including students unable to attend school, middle‐aged individuals unable to work, and the elderly unable to go out, experienced a decrease in emotional loneliness as they united with their families to cope with the challenges (Killgore et al. 2020a). This evidence leads to the formulation of the following hypotheses.

Hypothesis 2 *COVID‐19 fear has negative associations with family emotional loneliness (i), social loneliness (ii), romantic emotional loneliness (iii)*.

### 1.2 Depression, Loneliness and Satisfaction With Life

Earlier research typically showed a positive relationship between depression and loneliness (Erzen and Çikrikci 2018; Ge et al. 2017). Loneliness, a phenomenon individuals recognise and strive to overcome, experienced a shift during the COVID‐19 pandemic. The fear induced by the virus heightened individuals' perceived social support and concurrently reduced feelings of loneliness. However, this trend reverses for individuals experiencing depression, a severe psychological illness that adversely impacts emotions, thoughts, and behaviours (Conejero et al. 2018; Kupferberg et al. 2016; Rnic et al. 2016).

Depression leads to diminished motivation, increased sadness and fatigue, and a range of problems affecting satisfaction with life (Busch et al. 2016; Koçak 2021a; Peres et al. 2017). Consequently, depressed individuals often withdraw from establishing relationships with their existing and potential social environments (DiTommaso et al. 2007). Unless they receive support or actively work to overcome depression, their loneliness is likely to intensify. Given that the impact of the COVID‐19 disease affects individuals of all ages, varying levels of depression are evident across different segments of the population (Dahlberg et al. 2022; DiTommaso et al. 2007). Studies (e.g., Lasgaard et al. 2011) revealed the connections between social and family loneliness with depression, while social and romantic loneliness were associated with social phobia (Lasgaard et al. 2011). Depressed individuals, therefore, have the potential to influence social, family, and romantic dimensions of loneliness, subsequently negatively impacting their overall life satisfaction (Gigantesco et al. 2019; Koçak 2021b; Mahmoud et al. 2012; Tomás et al. 2019). This context lays the foundation for the following hypotheses.

Hypothesis 3 *Depression has a positive relationship with family emotional loneliness (i), social loneliness (ii), and romantic emotional loneliness (iii)*.

Hypothesis 4 *Depression has a negative relationship with satisfaction with life*.

Available evidence shows that loneliness is associated with a reduction in satisfaction with life (Bakalım and Muyan 2020; Clair et al. 2021; Kapıkıran 2016; Koçak 2021a; Ozben 2013; Şimşek et al. 2021; Wang and Yao 2020). However, there exists a research gap regarding the predictive power of loneliness when considering its social, family, and romantic dimensions about life satisfaction. Some studies have attempted to address this gap by employing the social and emotional loneliness scale for adults to investigate how different dimensions of loneliness predict various outcomes (DiTommaso et al. 2016; Salimi 2011). For example, research revealed a negative relationship between all sub‐dimensions of loneliness and life satisfaction, with family emotional and romantic emotional loneliness emerging as stronger predictors than social loneliness (Bozorgpour and Salimi 2012; Salimi 2011). Similarly, studies conducted in different cultures, such as Turkish and Polish cultures, demonstrated negative relationships between social and emotional loneliness and satisfaction with life (Adamczyk and Ditommaso 2014; Akgül 2020). These findings form the basis for the following hypotheses.

Hypothesis 5 *Romantic emotional loneliness (i), social loneliness (ii), and family emotional loneliness (iii) have negative relationships with satisfaction with life*.

The existing literature revealed a negative association between COVID‐19 fear and social and emotional loneliness (Luchetti et al. 2020; Strizhitskaya et al. 2021). Individuals experiencing challenges posed by the COVID‐19 pandemic have paradoxically felt a heightened social and emotional closeness to their relatives (Killgore et al. 2020b). Consequently, COVID‐19 fear appears to diminish social and emotional loneliness. However, if effective solutions cannot be proposed to address the issues deriving from COVID‐19 fear and those at risk of infection cannot access social support from their immediate environment, coping becomes challenging, potentially leading to depression. The symptoms of depression exacerbate feelings of loneliness, driving individuals further away from their social circles. Hence, it is evident from the literature that when COVID‐19 fear, left unmitigated, transitions into depression, social and emotional loneliness tend to intensify. This establishes a mediating link via depression between COVID‐19 fear and social and emotional loneliness. Similarly, it is anticipated that loneliness will mediate the negative association between depression and satisfaction with life. Based on these reasonings, the following hypotheses were generated.

Hypothesis 6 *Depression has a mediator role between COVID‐19 fear and romantic emotional loneliness (i), social loneliness (ii), and family emotional loneliness (iii)*.

Hypothesis 7 *Family loneliness (i), social loneliness (ii), and romantic loneliness (iii) have a mediation effect on the relationship between COVID‐19 fear and satisfaction with life*.

Hypothesis 8 *Depression and family loneliness (i), depression and social loneliness (ii), and depression and romantic loneliness (iii) have serial mediation effects in the associations between COVID‐19 fear and satisfaction with life*.

Hypothesis 9 *Family loneliness (i), social loneliness (ii), and romantic loneliness (iii) have a mediation effect on the relationship between depression and satisfaction with life*.

Similar to wars and socio‐political changes, pandemics have caused significant influence over the economic and political fabric of societies. The COVID‐19 pandemic, in a similar vein, poses a threat to the economic well‐being of all societies at both the state and individual levels (Brodeur et al. 2021; Ceylan et al. 2020). Prolonged periods of restrictions, business closures, and escalating unemployment have introduced severe social and economic risks on a global scale. Numerous industry, service, and retail entities have been compelled to cease operations, facing unprecedented challenges (Brodeur et al. 2021; Vidya and Prabheesh 2020).

Despite government efforts to provide social and economic support, these measures have proven insufficient. Economic downturns in various sectors have translated into both tax losses for the state and income loss for individuals (Goodell 2020; Padhan and Prabheesh 2021). Global supply chain disruptions, alongside shrinking consumer demands, have prompted sectors to curtail investments and downsize their workforce (Davidescu et al. 2021; Goodell 2020; Vidya and Prabheesh 2020). Particularly vulnerable are low‐income and low‐skilled workers who face the constant threat of job loss (Deb et al. 2020; Kawohl and Nordt 2020).

Financial struggles within families have, in turn, caused the inability to meet the daily needs of university students. The stress experienced by middle‐aged workers and students working during the COVID‐19 pandemic has not been adequately alleviated, often evolving into prolonged periods of depression over time (Bocchino et al. 2021; Lee et al. 2021). Moreover, the literature indicated that during the COVID‐19 period, single and female individuals tended to experience higher levels of stress compared to their married counterparts (Khademian et al. 2021; Ustun 2021). However, conflicting findings also exist, with some studies reporting no significant differences between single and married individuals or between males and females (Khademian et al. 2021).

Hypothesis 10 *There are differences in the psychological consequences of COVID‐19 fear according to economic conditions, marital status and gender*.

## 2 Current Study

**FIGURE 1:**
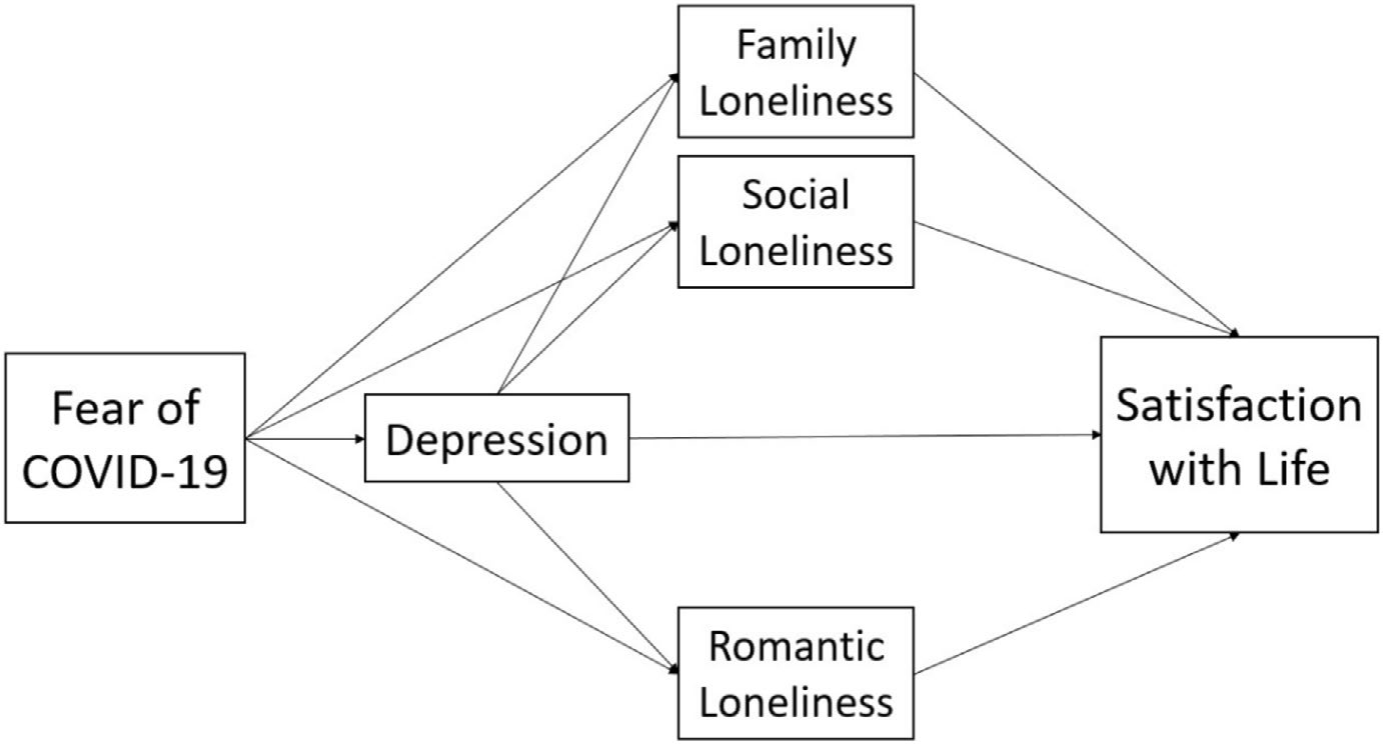
Conceptual model of the research for SEM analyses.

Based on the theoretical and empirical rationale outlined earlier, this study sought to explore the impact of COVID‐19 fear on depression, subsequently influencing various dimensions of loneliness—specifically family loneliness, social loneliness, and romantic loneliness—ultimately affecting individuals' levels of life satisfaction. To achieve this, the hypotheses formulated in the literature were visually presented in Figure 1, depicting the conceptual mediational model. In Figure 1, the conceptual model outlines direct, indirect, and serial mediation relations, providing a visual representation of the hypotheses to be empirically tested. This approach aimed to provide evidence regarding the mediating roles of depression and loneliness in the relationship between fear of COVID‐19 and life satisfaction during the COVID‐19 pandemic.

**FIGURE 2:**
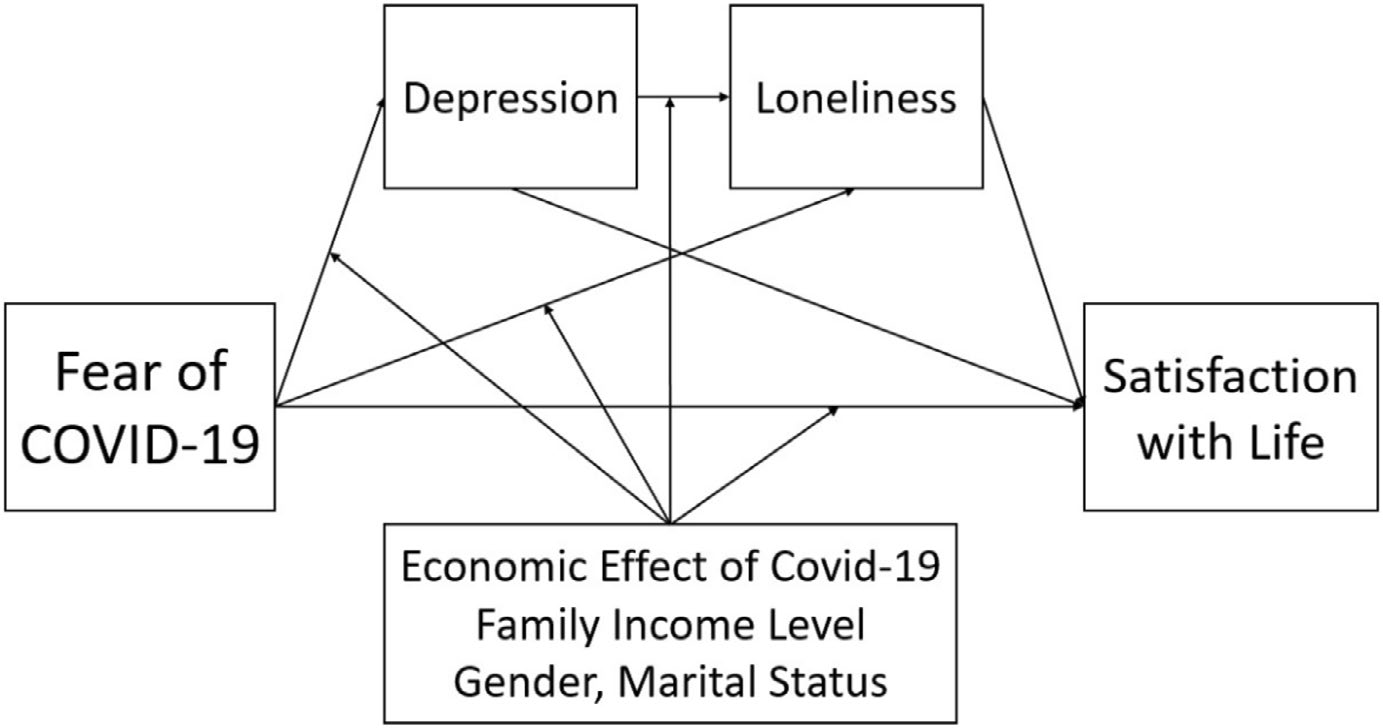
Conceptual model of the research for moderating analyses.

Furthermore, we aimed to test the moderating roles of economic effect, family income, gender, and marital status in the associations between the study variables. Figure 2 illustrates the model designed to test the moderator hypothesis in this study. The composite score for loneliness was used in this model. Key control variables included participants' age, gender, family income, marital status, and whether they were economically affected by COVID‐19 restrictions. These variables were incorporated into the model to ensure the potential influence of various demographic and economic factors on the emerging relationships between the study variables.

## 3 Method

### 3.1 Participants

The study employed a snowball sampling method to recruit participants. Inclusion criteria required individuals to be at least 15 years old, fluent in Turkish, and willing to participate voluntarily. Exclusion criteria included individuals who did not meet these requirements. Given the widespread susceptibility of individuals of all ages to the COVID‐19 disease, our study included a total of 1702 participants aged 15 and above (M = 26.10; SD = 9.24). The majority of participants were recruited through health science students, resulting in a predominantly female and youthful demographic. Among the participants, 68% were female, and 32% were male. The findings revealed that 21.1% of respondents reported no known infections among their families or relatives, while 63.7% had experienced a COVID‐19 infection, and 14.7% had died due to the disease. The demographic information of the participants is given in Table 1.

**TABLE 1:** Descriptive statistics.

Variable	Level	*N*	%	Mean	SD
Gender	Female	1158	68		
	Male	544	32		
Age				26.1	9.236
Marriage				1.92	0.272
	Married	408	24		
	Single	1270	74.6		
	Divorced	24	1.,4		
Education					
	Elementary	53	3.1		
	Middle	53	3.1		
	High school	320	18.8		
	University	1203	70.7		
	Master or PhD.	73	4.3		
Income				2.76	1.198
	Low	243	14.3		
	Lower middle	557	32.7		
	Middle	454	26.7		
	Upper middle	261	15.3		
	High	187	11		

### 3.2 Measures

**The COVID‐19 Fear Scale** (FCV‐19S), originally developed by Ahorsu et al. (2020) and adapted to Turkish by Bakioğlu et al. (2020), was used to measure fear related to COVID‐19 in this study. The FCV‐19S includes seven items. Responses were collected on a five‐point Likert scale, ranging from strongly disagree (1) to strongly agree (5). The reliability and validity of the adapted version were satisfactory. In our current study, the Cronbach's Alpha value for the FCV‐19S was 0.866.

**The Depression Anxiety Stress Scale‐21** (DASS‐21) was initially developed by Lovibond and Lovibond (1995) and adapted to Turkish by Yılmaz et al. (2017). The DASS‐21 assesses depression, stress, and anxiety with seven items each. The scale has a four‐point Likert scale ranging from 0 = not suitable for me to 3 = completely suitable for me. The depression sub‐factor, used in this study to measure the symptoms of depression, demonstrated a Cronbach's Alpha coefficient of 0.877.

**The Social and Emotional Loneliness Scale for Adults** (SELSA) used in our research was developed by DiTommaso and Spinner (1993), and its Turkish adaptation was conducted by Akgül in 2020. The SELSA comprises 15 items, divided into three sub‐dimensions: social loneliness, emotional loneliness, and romantic loneliness. Responses were collected on a seven‐point Likert scale, ranging from 1 (strongly disagree) to 7 (strongly agree). In the current study, the Cronbach Alpha values for the social, family, and romantic loneliness sub‐dimensions were 0.90, 0.89, and 0.87, respectively.

**The Satisfaction With Life Scale** (SWLS), originally developed by Diener et al. (1985) and adapted to Turkish by Dağlı and Baysal (2016), was used to measure life satisfaction. This SWLS consists of five items, and each item is rated on a seven‐point Likert scale ranging from strongly disagree (1) to strongly agree (7), with a Cronbach Alpha internal consistency coefficient of 0.88 in the Turkish adaptation. In our current study, the internal consistency value for the SWLS was 0.861.

### 3.3 Procedure

The research was designed as cross‐sectional and quantitative. The study followed a structured approach, with data collection conducted online to reach a diverse sample efficiently using the Survey Monkey online platform. After explaining why the research was conducted at the beginning of the online form, the consent of the participants was obtained. The study initially gathered data from a total of 1820 participants across various cities in Turkey. Following the completion, instances with missing responses were excluded, resulting in a final sample size of 1702. To uphold privacy, no personal information or IP numbers were collected, and participants retained the option to withdraw from the research at any point. The average response time for the survey was determined to be between 10 and 20 min. The research protocol adhered to the ethical standards outlined in the Declaration of Helsinki. The study protocol was reviewed and approved by Istanbul University—Cerrahpaşa (reference number: E‐74555795‐050.01.04‐546847).

## 4 Data Analyses

After the data were arranged in the SPSS program, confirmatory factor analysis was performed to determine the suitability of the measurement model and the construct validity of the factors using the IBM AMOS 24 program (Amos 2017). After determining the suitability of the measurement model, a new SPSS file was created by performing data imputation to make a preliminary analysis. Relevant analyses in SPSS were used for reliability, mean, standard deviation, skewness, and kurtosis values required to perform structural equation modelling path analysis. To see detailed indirect effects in structural equation modelling path analysis, the IndirectEffects Plug‐in developed by Gaskin was added to the AMOS 25 program (Gaskin and Lim 2018). Structural equation path analysis was performed with 2000 bootstraps and a 95% confidence interval. Process Macro Plug‐in SPSS for moderator analyses by using Model 92 with 5000 bootstraps and a 95% confidence interval (Hayes and Rockwood 2020) and simple slope templates to produce two‐way interaction graphics designed by Dawson were used in the study (Dawson 2014).

## 5 Results

### 5.1 Preliminary Analysis

The study employed confirmatory factor analysis using the IBM AMOS 24 statistical program to evaluate the proposed measurement model. After implementing five covariances identified in the modification indices, model fit values were found to be consistent with Kline's recommended criteria (CIMIN/df < 5, TLI, IFI, CFI, GFI, CFI, NFI > 0.90, RMSEA < 0.80) (Kline 2016). The measurement model exhibited high values (see Table 2).

**TABLE 2:** Measurement model and SEM path analysis values.

Measure	Measurement values	Path values	Cut‐off criteria
CMIN/DF	3278	3.705	< 5
CFI	0.965	0.958	> 0.90
SRMR	0.048	0.0571	< 0.08
RMSEA	0.037	0.040	< 0.08
NFI	0.95	0.943	> 0.90
GFI	0.948	0.940	> 0.90
IFI	0.965	0.958	> 0.90
AGFI	0.938	0.928	> 0.90
TLI	0.96	0.952	> 0.90

After determining the suitability of the measurement model, a new SPSS file was produced with the factors by performing data imputation. Correlations, averages, standard deviations, Cronbach's alpha, skewness, and kurtosis values of the factors were determined in the analyses (see Table 3). It was understood that all of the correlation values of the factors were significant at the *p* < 0.01 level. Cronbach's Alpha values were above 0.75, and skewness and kurtosis values were among the normal distribution values.

**TABLE 3:** Correlations, means, standard deviations, skewness, kurtosis.

		1	2	3	4	5	6
1	COVID‐19 fear	1					
2	Depression	0.096**	1				
3	Family loneliness	−0.092**	0.531**	1			
4	Social loneliness	−0.065**	0.453**	0.631**	1		
5	Romantic loneliness	−0.149**	0.284**	0.188**	0.087**	1	
6	Satisfaction with Life	0.034	−0.637**	−0.476**	−0.427**	−0.260**	1
	Cronbach's alpha	0.866	0.877	0.831	0.775	0.822	0.861
	Mean	2.192	1.342	1.635	2.041	4.138	1.675
	Standard deviation	0.772	0.455	0.886	0.901	2.212	0.664
	Skewness	0.024	0.819	1.217	0.667	−0.253	−0.058
	Kurtosis	−0.632	0.776	1.099	−0.028	−1.601	−0.522

** *p* < 0.01.

### 5.2 Testing the Mediational Model

Following the confirmation of the appropriateness of the measurement model ascertained through normality and reliability tests, an SEM path analysis was conducted. As presented in the results, it showed statistically significant direct associations as well as indirect relationships between the study variables (see Table 4).

**TABLE 4:** Direct and indirect effects.

Paths	*β*	*B*	SE	*p*
COVID‐19 fear → Depression	0,086	0,049	0,016	**
COVID‐19 fear → Family loneliness	−0,131	−0,146	0,029	***
COVID‐19 fear → Social loneliness	−0,094	−0,110	0,034	**
COVID‐19 fear → Romantic loneliness	−0,163	−0,439	0,071	***
Depression → Family loneliness	0,497	0,970	0,07	***
Depression → Social loneliness	0,425	0,866	0,073	***
Depression → Romantic loneliness	0,280	1323	0,125	***
Depression → Satisfaction with life	−0,452	−0,649	0,05	***
Romantic loneliness → Satisfaction with life	−0,091	−0,028	0,007	***
Social loneliness → Satisfaction with life	−0,105	−0,074	0,02	***
Family loneliness → Satisfaction with life	−0,145	−0,107	0,021	***
COVID‐19 fear → Depression → Family loneliness	0,043	0,047	0,018	**
COVID‐19 fear → Depression → Family loneliness → SwL	0,043	−0,005	0,002	**
COVID‐19 fear → Depression → Social loneliness	0,036	0,042	0,016	**
COVID‐19 fear → Depression → Social loneliness → SwL	0,036	−0,003	0,002	**
COVID‐19 fear → Depression → Romantic loneliness	0,024	0,065	0,024	**
COVID‐19 fear → Depression → Romantic loneliness → SwL	0,024	−0,002	0,001	**
COVID‐19 fear → Depression → Satisfaction with life	−0,039	−0,032	0,012	**
COVID‐19 fear → Family loneliness → Satisfaction with life	0,019	0,016	0,005	***
COVID‐19 fear → Social loneliness → Satisfaction with life	0,010	0,008	0,004	**
COVID‐19 fear → Romantic loneliness → Satisfaction with life	0,015	0,012	0,004	***
Depression → Family loneliness → Satisfaction with life	−0,072	−0,103	0,022	**
Depression → Social loneliness → Satisfaction with life	−0,045	−0,064	0,021	**
Depression → Romantic loneliness → Satisfaction with life	−0,026	−0,037	0,01	***

Abbreviations: *B* = estimate; SE = standard error; SwL = satisfaction with life; *β* = standardised estimate.

** *p* < 0.01.

*** *p* < 0.001.

The findings of the mediation analysis demonstrated that COVID‐19 fear was associated with increased depression and a decrease in family, social, and romantic loneliness. Depression and various forms of loneliness—social, family emotional, and romantic emotional—exhibited negative associations with life satisfaction. The study also identified that depression served as a mediator in the relationships between COVID‐19 fear and the sub‐factors of loneliness: family loneliness, social loneliness, and romantic loneliness. Additionally, the mediation effect of these loneliness sub‐factors was observed in the relationship between depression and life satisfaction. The analysis showed that 38% of the variance in life satisfaction, 25% in family loneliness, 18% in social loneliness, and 10% in romantic loneliness could be accounted for by the studied variables. The findings predominantly support the hypotheses posited in the study (see Table 5).

**TABLE 5:** Hypotheses results.

Paths	Hypothesis	
	Support	
COVID‐19 fear → Depression	H1	Yes
COVID‐19 fear → Family loneliness	H2i	Yes
COVID‐19 fear → Social loneliness	H2ii	Yes
COVID‐19 fear → Romantic loneliness	H2iii	Yes
Depression → Family loneliness	H3i	Yes
Depression → Social loneliness	H3ii	Yes
Depression → Romantic loneliness	H3iii	Yes
Depression → Satisfaction with life	H4	Yes
Romantic loneliness → Satisfaction with life	H5i	Yes
Social loneliness → Satisfaction with life	H5ii	Yes
Family loneliness → Satisfaction with life	H5iii	Yes
COVID‐19 fear → Depression → Romantic loneliness	H6i	Yes
COVID‐19 fear → Depression → Social Loneliness	H6ii	Yes
COVID‐19 fear → Depression → Family loneliness	H6iii	Yes
COVID‐19 fear → Family loneliness → Satisfaction with life	H7i	Yes
COVID‐19 fear → Social Loneliness → Satisfaction with Life	H7ii	Yes
COVID‐19 fear → Romantic loneliness → Satisfaction with life	H7iii	Yes
COVID‐19 fear → Depression → Romantic loneliness → SwL	H8i	Yes
COVID‐19 fear → Depression → Social loneliness → SwL	H8ii	Yes
COVID‐19 fear → Depression → Family loneliness → SwL	H8iii	Yes
Depression → Family loneliness → Satisfaction with life	H9i	Yes
Depression → Social loneliness → Satisfaction with life	H9ii	Yes
Depression → Romantic loneliness → Satisfaction with life	H9iii	Yes
COVID‐19 fear → Depression → Loneliness → SwL Moderated by Economic Effect of COVID‐19, Family income, Marital status, and Gender	H10	Yes

#### 5.2.1 Testing the Moderation Model

We investigated the moderating roles of the economic impact of COVID‐19, family income, and gender in the associations between COVID‐19 fear, depression, and satisfaction with life. The analyses were conducted by creating a new interaction variable, which resulted from multiplying the participants' reported economic impact of COVID‐19 restrictions and their level of COVID‐19 fear (economic effect of COVID‐19 × COVID‐19 fear).

**FIGURE 3:**
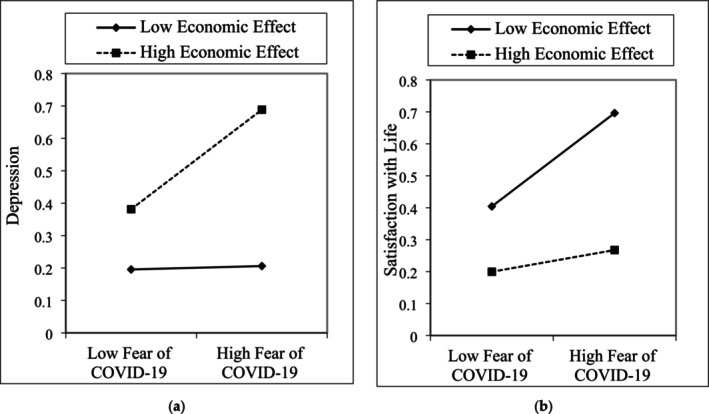
Moderation role of economic effect between COVID‐19 and depression (a), and SwL (b).

The moderation analysis, executed with Model 62 in the Process Macro Plug‐in for SPSS, revealed a significant moderating role of the economic impact of COVID‐19 in the relationship between COVID‐19 fear and depression (*B* = 0.0855, *p* < 0.01). The results indicated that the association between COVID‐19 fear and depression was lower in individuals with a low economic impact of COVID‐19 compared to those with a high economic impact. Consequently, the impact of COVID‐19 fear on depression was more substantial for individuals more economically affected by the restrictions than for those less affected (see Figure 3a).

Furthermore, the moderation analysis identified a moderating role of the economic impact of COVID‐19 in the relationship between COVID‐19 fear and satisfaction with life (B = −0.0646, *p* < 0.05). The relationship between COVID‐19 fear and satisfaction with life was found to be lower in individuals with a high economic impact of COVID‐19, while it was higher in those less affected (see Figure 3b). The level of satisfaction with life tends to be lower for those who experience a greater economic impact from COVID‐19 restrictions.

**FIGURE 4:**
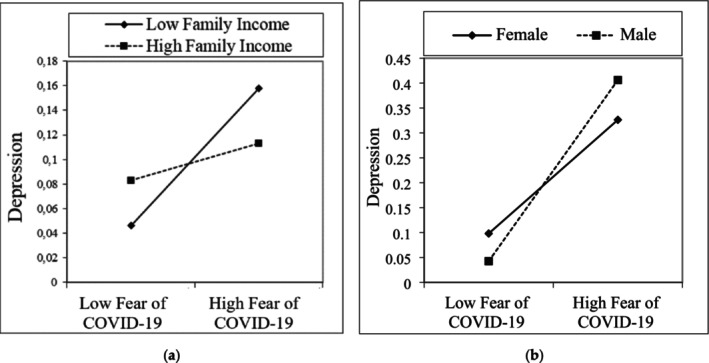
Moderation role of family income (a) and gender (b) between Covid‐19 and depression.

In addition, a new interaction variable was formulated by multiplying the participants' reported family income levels and their level of COVID‐19 fear (family income × COVID‐19 fear). The moderator analysis revealed a significant association, indicating that COVID‐19 fear heightened depression, with this effect being higher in individuals with low family income compared to those with high family income (B = −0.0222, *p* < 0.05), (see Figure 4a). This suggests that families with lower income levels are more susceptible and exhibit a higher propensity for depression triggered by COVID‐19 fear. Furthermore, a different interaction variable was created by multiplying the participants' reported gender and their level of COVID‐19 fear (gender × COVID‐19 fear). The moderator analysis showed that the impact of COVID‐19 fear on depression was higher in males than in females (*B* = 0.0677, *p* < 0.05) (see Figure 4b). This implies that males are more vulnerable and significantly influenced by the effect of COVID‐19 fear on depression compared to females.

**FIGURE 5:**
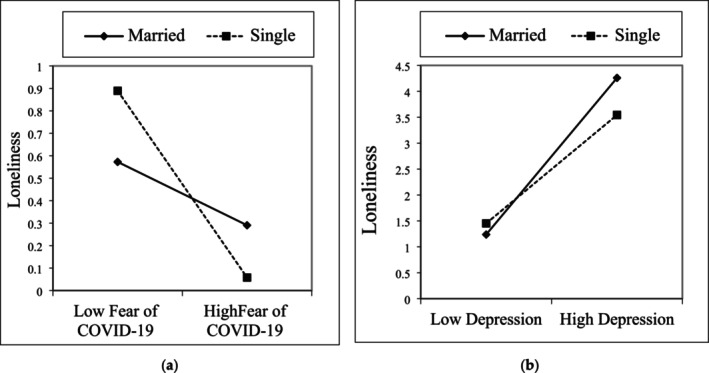
Moderation role of marital status between COVID‐19 (a), depression (b) and loneliness.

Another interaction variable was generated by multiplying the participants' reported marital status and their level of COVID‐19 fear (marital status × COVID‐19 fear). The moderator analysis revealed that COVID‐19 fear was associated with a reduction in loneliness among married individuals, albeit this reduction was lower in singles (*B* = −0.1583, *p* < 0.01), (see Figure 5a). Subsequently, an additional interaction variable was created by multiplying the participants' reported marital status and their level of depression (marital status × depression). The moderator analysis indicated that the impact of depression on loneliness was more significant in married individuals compared to singles (*B* = −0.4097, *p* < 0.001) (see Figure 5b).

## 6 Discussion

In the current study, we investigated the relationship between individuals' experience of COVID‐19 fear and their satisfaction with life. Additionally, we explored how depression, family, social, and romantic loneliness mediated this association. We also examined the moderating roles of the economic impact of COVID‐19, family income, marital status, and gender in this relationship. The results typically provided support for the hypotheses of this study in terms of direct effects, indirect effects, and interaction effects.

### 6.1 Direct Effects Between the Variables

The study affirmed the positive relationship between COVID‐19 fear and depression (H1), as well as the negative associations with three sub‐factors of loneliness—family loneliness (H2i), social loneliness (H2ii), and romantic loneliness (H2iii). Therefore, hypotheses H1, and H2i–iii were supported. The global spread of the COVID‐19 pandemic, driven by widespread societal mobility (Borkowski et al. 2021; Saha et al. 2020), prompted preventive measures that negatively impacted various facets of societies, ranging from economics, social dynamics, health, and education to employment (Yıldırım and Özaslan 2022). This, in turn, contributed to increased levels of psychological problems such as stress, anxiety, and depression among individuals (Abuselidze and Mamaladze 2020; Jain et al. 2020; Koçak 2021b; Kumar et al. 2021; Lakhan et al. 2020). Additionally, the prolonged enforcement of preventive measures, particularly affecting young and middle‐aged working individuals, intensified symptoms of depression among people (Giorgi et al. 2020; Koçak 2021b; Koçak et al. 2021). The escalating severity of measures in response to the increasing COVID‐19‐related fatalities, extensively covered in daily media reports, further exacerbated the adverse effects on people (Bendau et al. 2021; Choi et al. 2020; Liu and Liu 2020). In light of these negative circumstances, it is evident that COVID‐19 fear contributes to an increase in individuals' depression.

The analysis revealed a negative relationship between COVID‐19 fear and loneliness sub‐factors, indicating that COVID‐19 fear diminishes individuals' inclination toward negative thoughts and situations related to loneliness. These findings contrasted with the usual positive association between psychological problems (e.g., fear) and loneliness in the extant literature. The findings show a distinctive pattern during extraordinary circumstances, such as the COVID‐19 pandemic. The existing literature recognises a difference between the sense of loneliness induced by the COVID‐19 pandemic and that experienced in other periods (Killgore et al. 2020a; Luchetti et al. 2020; Strizhitskaya et al. 2021). The challenges posed by the pandemic required individuals to confront unforeseen circumstances, prompting a desire to strengthen emotional connections with family, relatives, and close friends. Consequently, individuals experienced a reduction in loneliness during the COVID‐19 period as they actively strengthened emotional and social bonds with their immediate social circles (Campbell 2020; Yoosefi Lebni et al. 2021). Thus, the challenges of the COVID‐19 period led individuals to reevaluate and enhance their relationships with family, relatives, close friends, and neighbours as a collective strategy to cope with these unprecedented challenges.

The findings also revealed a positive relationship between depression and three dimensions of loneliness—family, social, and romantic—along with a negative relationship with satisfaction with life. Consequently, hypotheses H3i–iii, and H4 have been supported. Given that depression is a severe psychological problem, it exerts a detrimental impact on individuals' attitudes and thoughts (Conejero et al. 2018; Kupferberg et al. 2016; Rnic et al. 2016). Consistent with the existing literature, the current study found that depression positively predicts loneliness (Busch et al. 2016; Koçak 2021a; Peres et al. 2017).

The majority of participants were young and literature indicated that young people, especially during the COVID‐19 pandemic, experienced heightened future anxiety (Yıldırım et al. 2023) due to disruptions in education, socialisation, work, and career planning, leading to manifestations of depression and loneliness (Cruwys et al. 2021; Okruszek et al. 2020; Varma et al. 2021). In line with this, the study supported hypothesis 4, revealing a significant negative relationship between depression and satisfaction with life. The literature highlights that stress, anxiety, and depression are associated with decreased levels of quality of life and satisfaction with life (Huang and Zhang 2022; Lim and Putnam 2010; Zheng et al. 2019). Thus, the current study emphasises that the elevated negative symptoms, such as depression experienced by young individuals during the COVID‐19 pandemic period, have contributed to a reduction in their satisfaction with life.

Furthermore, in this study, hypothesis H5i–iii received support, as it was established that romantic, social, and family dimensions of loneliness exhibited significant negative associations with satisfaction with life. The findings are consistent with previous research indicating negative associations between loneliness and satisfaction with life (Adamczyk and Ditommaso 2014; Clair et al. 2021; Wang and Yao 2020). Consistent results have also been reported in studies conducted in Türkiye (Akgül 2020; Bakalım and Muyan 2020; Kapıkıran 2016; Ozben 2013; Şimşek et al. 2021). Importantly, the present study makes a significant contribution to the field by examining the associations between loneliness, with its diverse dimensions of family, romantic, and social, and satisfaction with life. While existing literature acknowledges the negative impact of loneliness on life satisfaction, few studies explore the dimensions of loneliness in this context. The focus on family, romantic, and social dimensions in this study enhances our understanding of the relationship between loneliness sub‐factors and life satisfaction. Particularly, emotional and social loneliness can hinder the development of fulfilling and high‐quality social relationships over an extended period, ultimately diminishing individuals' satisfaction with life (Hawkley and Cacioppo 2010; Heu et al. 2021; Lee and Ishii‐Kuntz 2016; Mushtaq et al. 2014).

#### 6.1.1 Indirect Effects Between the Variables

We conducted indirect relationships between the variables through the establishment of hypotheses. Initially, the study examined the mediating role of depression in the relationship between COVID‐19 fear and the sub‐factors of loneliness: romantic, social, and family. Each mediation test yielded significant results. In all mediation analyses, the mediating variables demonstrated significant associations with the independent and dependent variables. In addition, the mediation analysis revealed that the confidence interval values did not overlap with zero, providing support for the hypotheses H6i–iii, H7i–iii, H8i–iii, and H9i–iii.

As individuals experienced the challenges posed by the pandemic, a significant shift toward developing emotional bonds with family, relatives, and close friends was observed (Killgore et al. 2020b; Luchetti et al. 2020). However, the psychological consequences of COVID‐19 fear could lead to depressive symptoms, which in turn diminish satisfaction with life. That is, COVID‐19 fear triggered depressive behaviours and social isolation, culminating in heightened levels of loneliness, which in turn reduced life satisfaction. In line with existing literature, the study revealed that unmitigated COVID‐19 fear leads to depression related to increased social and emotional loneliness, alongside a decline in satisfaction with life levels.

#### 6.1.2 Interaction Effects Between the Variables

The study provided evidence about the moderator roles of the economic impact of COVID‐19, family income, marital status, and gender in the associations between COVID‐19 fear, depression, loneliness, and satisfaction with life. Thus, the moderator hypothesis (H10) of this research gained empirical support. The findings suggest the significant influence of the economy on individuals' well‐being during the COVID‐19 pandemic, particularly for families facing economic challenges. Low‐income families experienced a higher impact of COVID‐19 fear on depression compared to their higher income counterparts.

The economic impact of pandemic‐related measures exacerbated existing financial hardships, especially for low‐income and unskilled workers, including university students (Brodeur et al. 2021; Ceylan et al. 2020; Goodell 2020; Vidya and Prabheesh 2020). Notably, the study revealed that COVID‐19 fear heightened depression more significantly among men than women, contrary to the trend in the literature, which suggests that women generally experience more psychological problems during the pandemic (Bocchino et al. 2021; Lee et al. 2021). The higher labour force participation rate of men in Turkish society may contribute to increased stress and depression, given their multifaceted responsibilities, including supporting their families.

The study revealed a significant gender difference, indicating a higher increase in depression among men than women due to COVID‐19 fear, contrary to prevalent findings in the literature highlighting more psychological challenges for women during the pandemic (Khademian et al. 2021; Ustun 2021). This discrepancy might be attributed to the unique labour force dynamics in Turkish society, where men exhibit double the participation rate compared to women. The increased stress and depression among men could emerge from the added responsibilities they shoulder, such as employment and supporting their families during the COVID‐19 period.

Interestingly, the findings suggested a dual effect of COVID‐19 fear: a reduction in loneliness but a concurrent increase in depression. The pandemic necessitated extended periods of home confinement, prompting individuals to spend more time with their families and enhance familial bonds. Consequently, being married during the pandemic emerged as a mitigating factor against loneliness, with married individuals drawing support from within their families. Conversely, singles actively sought increased connections with family, relatives, and close friends to alleviate loneliness during this period. However, the impact of depression presented a contrasting picture, indicating that married individuals experienced heightened loneliness compared to their single counterparts during the pandemic. This paradoxical finding suggested that the responsibilities associated with marriage might incline depressed individuals toward a preference for solitude.

## 7 Strengths of the Study

This study has several strengths. First, the large sample size (*N* = 1702) enhances the reliability and generalisability of the findings within the Turkish population. Second, the study examines multiple psychological factors, including fear of COVID‐19, depression, different types of loneliness (social, emotional, and romantic), and life satisfaction, providing a better understanding of psychological health during the pandemic. Third, by employing mediation and moderation analyses, the study moves beyond simple associations to explore underlying mechanisms and contextual influences, presenting deeper insights into the relationships among these variables. Specifically, the findings highlight the mediating role of depression in the link between COVID‐19 fear and loneliness while also identifying key moderating factors such as economic impact, family income, and gender. These results have practical implications for mental health interventions and policy development, particularly in identifying and supporting vulnerable groups affected by the pandemic.

## 8 Limitations of the Study

This study is not without limitations. First, conducting this research during the COVID‐19 pandemic presented challenges, particularly in the absence of face‐to‐face interviews, limiting our ability to interpret participants' facial expressions, thoughts, and responses. Second, the exclusive focus on the COVID‐19 pandemic in Türkiye prohibits direct comparisons with other periods or countries. Furthermore, the predominantly youthful composition of the sample restricts the generalisability of findings to broader society. Additionally, the cross‐sectional, online, and quantitative nature of the study warrants caution against drawing inappropriate comparisons with studies using different methodologies.

### 8.1 Implications

This study highlights the psychological impact of the COVID‐19 pandemic by emphasising the significant role of fear, depression, and loneliness in influencing individuals' life satisfaction. It suggests how the fear of COVID‐19, although not directly lowering life satisfaction, indirectly diminishes it through depression and loneliness, particularly in vulnerable demographic groups such as young people, middle‐aged individuals, and those from lower‐income families. The economic impacts of COVID‐19 restrictions, including job loss, financial instability, and isolation, have exacerbated these psychological challenges. The findings emphasise the need for policies tailored to support disadvantaged populations, particularly the youth, during such unprecedented times. Social, educational, and economic interventions must be designed with an understanding of the psychological dynamics revealed in this study, ensuring they are adaptable to evolving societal needs. Targeted measures in sectors such as education, healthcare, and social support are necessary to mitigate the psychological effects of global crises like pandemics and to ensure the well‐being of those most affected.

## 9 Conclusion

In conclusion, this study contributes to the understanding of the psychological factors that mediate and moderate the relationship between COVID‐19 fear and life satisfaction. It provides evidence about the relationships between fear, depression, loneliness, and socio‐economic factors, highlighting how these elements influence life satisfaction during times of crisis. The findings suggest that addressing the mental health challenges exacerbated by COVID‐19 requires a comprehensive, multidimensional approach that considers not only psychological interventions but also economic and social support systems. Given the ongoing impact of the pandemic and its socio‐economic consequences, policymakers and public health officials must adapt their strategies to the evolving needs of vulnerable populations, ensuring that these interventions remain relevant and effective in the face of future crises.

## Author Contributions

Study conception/design: O.K. Methodology: O.K., M.Y. Data collection: O.K. Formal analysis: O.K. Supervision: M.Y. Writing – original draft: O.K., M.Y. Review and editing: O.K., M.Y., O.M.Ş., O.Ç. All authors have read and agreed to the published version of the manuscript.

## Ethics Statement

All procedures performed in studies involving human participants were in accordance with the ethical standards of the institutional and national research committee and with the 1964 Helsinki Declaration and its later amendments or comparable ethical standards. The study protocol was reviewed and approved by Istanbul University—Cerrahpaşa (reference number: E‐74555795‐050.01.04‐546847).

## Consent

Consent was obtained from all participants included in the study.

## Conflicts of Interest

The authors declare no conflicts of interest.

## Data Availability

The data supporting this study's findings are available from the corresponding author upon reasonable request.
